# A Comprehensive Study on Processing Ti–6Al–4V ELI with High Power EDM

**DOI:** 10.3390/ma14020303

**Published:** 2021-01-08

**Authors:** Panagiotis Karmiris-Obratański, Emmanouil L. Papazoglou, Beata Leszczyńska-Madej, Krzysztof Zagórski, Angelos P. Markopoulos

**Affiliations:** 1 Laboratory of Manufacturing Technology, School of Mechanical Engineering, National Technical University of Athens, 15780 Athens, Greece; mlpapazoglou@mail.ntua.gr; 2 Department of Manufacturing Systems, Faculty of Mechanical Engineering and Robotics, AGH University of Science and Technology, 30-059 Cracow, Poland; zagkrzys@agh.edu.pl; 3 Department of Materials Science, Faculty of Non-Ferrous Metals, Non-Ferrous Metals Engineering, AGH University of Science and Technology, 30-059 Cracow, Poland; bleszcz@agh.edu.pl

**Keywords:** EDM, titanium alloy, machining performances, white layer, heat affected zone

## Abstract

Electrical Discharge Machining (EDM) consists of a non-conventional machining process, which is widely used in modern industry, and especially in machining hard-to-cut materials. By employing EDM, complex shapes and geometries can be produced, with high dimensional accuracy. Titanium alloys, due to their unique inherent properties, are extensively utilized in high end applications. Nevertheless, they suffer from poor machinability, and thus, EDM is commonly employed for their machining. The current study presents an experimental investigation regarding the process of Ti–6Al–4V ELI with high power EDM, using a graphite electrode. Control parameters were the pulse-on current (I_p_) and time (T_on_), while Machining performances were estimated in terms of Material Removal Rate (MRR), Tool Material Removal Rate (TMRR), and Tool Wear Ratio (TWR). The machined Surface Roughness was calculated according to the Ra and the Rt values, by following the ISO 25178-2 standards. Furthermore, the EDMed surfaces were observed under optical and SEM microscopy, while their cross sections were also studied in order the Average White Layer Thickness (AWLT) and the Heat Affected Zone (HAZ) to be measured. Finally, for the aforementioned indexes, Analysis Of Variance was performed, whilst for the MRR and TMRR, based on the Response Surface Method (RSM), semi-empirical correlations were presented. The scope of the current paper is, through a series of experiments and by employing statistical tools, to present how two main machining parameters, i.e., pulse-on current and time, affect major machining performance indexes and the surface roughness.

## 1. Introduction

Electro Discharge Machining (EDM) is a technologically advanced, high precision, non-conventional machining process. Conceptually, it is based on the use of erosion that accompanies electric discharges occurring between an electrode and a workpiece, both of which are submerged into a dielectric fluid [1]. By utilizing EDM, any electrically conductive material can be machined in complex shapes and geometries, in high dimensional accuracy, regardless of its mechanical properties. A pulsed voltage difference is applied between the workpiece and the working electrode, and under specific conditions (combinations of tool-electrode materials, voltage, and servo gap) a plasma channel is formed, with very high electromagnetic energy densities (up to 1014 W/m^2^). Topically, extremely high temperatures are developed, i.e., 6000–12000 °C, resulting in the melt and evaporation of material, from both the workpiece and the tool electrode [2,3,4,5,6]. Based on that material removal mechanism, difficult-to-cut materials can be machined, in an efficient and economically feasible way [7]. Another inherent advantage of the EDM process is the absence of the residual stresses due to the non-contact nature of the method, since there is no physical contact of the working electrode with the workpiece, and hence, no cutting forces are developed [8]. EDM is widely used in implants, molds, and tool manufacturing industries, as well in automotive and aerospace ones.

EDM is a complex, multiparameter process, including machining parameters like the pulse-on current (I_p_), the pulse-on time (T_on_), the duty factor (η), the applied voltage (V) and polarity, the flushing pressure, and the gap in-between the working electrode and the workpiece. Moreover, it is strongly affected by non-machining parameters, namely, by the electrode and workpiece material, and the type of the dielectric fluid (hydrocarbon oil or distilled water). Different materials have been tested and utilized as electrodes, with copper, graphite, tungsten, and composite materials produced with powder metallurgy being the most commonly used [9,10]. The machining performances are evaluated in terms of Material Removal Rate (MRR), Tool Wear Ratio (TWR), and the obtained Surface Quality (SQ) and Surface Topography (ST). Research is focus on maximizing of the MRR, and minimizing TWR, achieving machining efficiency and retaining high level of precision and geometrical accuracy. Moreover, and since EDM is commonly used in high-end applications, the SQ is a crucial parameter [11,12,13,14,15,16].

Titanium alloys, due to their unique properties, find extensive use in a wide range of modern applications, including the aerospace, automobile, and medical industries. They own a high strength at a low to moderate temperature, a superior strength to weigh ratio, excellent corrosion and wear resistance, fatigue durability, and high biocompatibility [17,18]. On the other hand, titanium alloys, due to their low thermal conductivity, high chemical reactivity, and low elasticity modulus, suffer from poor machinability, rendering them as hard-to-cut materials. Hence, non-conventional machining processes, like EDM, are often utilized in their machining [19,20].

Extensive research has been conducted regarding the machining of titanium alloys with EDM. Cherng Lin et al. [21] presented an experimental study concerning ultrasonic-assisted EDM of titanium Grade 5, by using distilled water and kerosine as dialectic fluids. From the experimental results they deduced that the ultra-sonic assistance improved the EDM efficiency by increasing the MRR. Hasçalık and Caydas [22,23] also conducted an experimental study on machining titanium Grade 5 with EDM, comparing the use of four different electrode materials, namely copper (normal and cryogenically treated), tungsten, and graphite. The machining performances were estimated in terms of MRR, the WL formation and its characteristics, and the crack density. It was concluded that the cryogenically treated electrode performed better compared to the other ones, offering an increased MRR, better surface finish, increased WL hardness, and related less Surface Crack Density (SCD). Fonda et al. [3] studied the effect of thermal and electrical properties of titanium Grade 5 on EDM productivity, while Klocke et al. [20] investigated the economic aspects and technological strategies in roughing machining of titanium Grade 5. It was deduced that EDM can be a competitive and feasible machining method in manufacturing, especially for small batches. Kumar et al. [24] developed and applied a hybrid Taguchi—Artificial Neural Network (ANN) to predict the Surface Roughness of cryogenically treated titanium alloys machined with EDM. Authors concluded that the machined SR is mainly affected by the pulse-on current, followed by the pulse-on time and the duty factor. Wang et al. [25] researched the influence of dielectrics’ characteristics on the machining of titanium TC4 alloy with EDM. Moreover, a compound dielectric was developed, achieving up to 500% higher MRR compared to the kerosine use, and an approximately 27% lower TWR. Mower [26] inquired into the fatigue strength degradation of the Ti–6Al–4V alloy after its machining with EDM. Its fatigue strength reduced between 15% and 30%, due to the formation of the recast layer and the increased surface roughness. Several authors reported the feasibility of using negative polarity to machine titanium alloys during EDM. Namely, Khan et al. [27,28] studied the effect of polarity in machining Ti–5Al–2.5Sn alloy with EDM, utilizing copper, copper-tungsten, and graphite electrodes. It was concluded that using negative polarity, the SR was almost doubled compared to the employment of positive polarity, and that the use of graphite electrode results in a better surface finish. Nair et al. [29] carried out experiments on Ti6Al4V, also with negative polarity, to investigate the impact of the machining parameters, i.e., T_on_, I_p_, and V, on the Surface Integrity and the MRR. It was deduced that higher pulse-on times and currents lead to increased MRR, Average White Layer Thickness (AWLT), and lower surface quality. In the work of Parkash et al. [30], the machining of Ti–35Nb–7Ta–5Zr β-titanium alloy was investigated, using hydrocarbon oil with a silicon powder additive as a dielectric medium. The machining performances were estimated in terms of MRR, TWR, WL, and SR, concluding that the silicon as a powder additive in dielectric fluid significantly improved the surface quality, while the MRR and the TWR were enhanced and reduced respectively. Ahmed et al. [31,32] studied the impact of four different electrode materials (copper, brass, aluminum, and graphite) on the EDM performance parameters (i.e., MRR and TWR) and the surface integrity. For the experiments, negative polarity was utilized, and it was concluded that graphite electrode offers higher MRR compared to the other ones, while the aluminum electrode resulted in the lowest SR. Finally, Farooq et al. [33] utilized Si Powder Mixed EDM (PWEDM) to modify the surface of a Ti–6Al–4V ELI (Extra Low Interstitial) alloy, while Bui et al. [34] investigated the deposition of silver on surfaces machined with EDM. The significance of detailed and in-depth research concerning the machining of different alloys with EDM, even if they belong in the same alloy class, is emphasized by Sen et al. [35] who studied how the B addition in Ti–6Al–4V alloys affect their machinability with EDM.

The scope of the current paper, in the relevant field of machining titanium alloys with EDM, is to present a comprehensive study regarding how the main machining parameters, i.e., the pulse-on current and pulse-on time, affect the process. In a series of experiments that were conducted, Ti–6Al–4V ELI was machined with high-power EDM by utilizing a graphite electrode. The obtained results, along with their subsequent statistical analysis, provide useful data, which can be utilized not only for further research purposes, but in a more applicable way too. The machining performances were estimated with regards to the Material Removal Rate (MRR), Tool Material Removal Rate (TMRR), and the Tool Wear Ratio (TWR), while the machined SR was measured in terms of mean roughness (Ra), and the maximum peak to valley height (Rz) by following the ISO 25178-2 standards. Moreover, the cross-sections were observed under optical microscope in order the AWLT and the Heat Affected Zone (HAZ) to be measured, while SEM microscopy was utilized to study the machined surfaces. Finally, for all the aforementioned indexes, Analysis Of Variance (ANOVA) was performed, and based on the Response Surface Method (RSM), semi-empirical correlations between the machining parameters and the MRR and TMRR were proposed.

## 2. Materials and Methods

**Figure 1:**
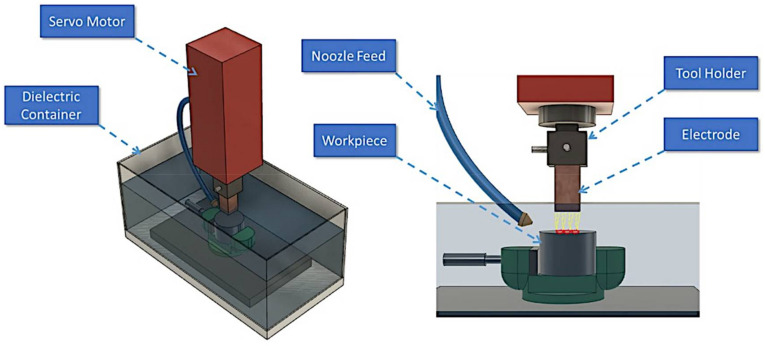
Graphical illustration of the experimental setup.

In the current experimental study, a 47 mm Ti–6AL–4V ELI (Grade 23) rode, cutoff in slices of 10mm, was used as a workpiece. Titanium Grade 23 is an alpha-plus-beta phase alloy, widely used in biomedical and aerospace industries. The experiments conducted utilizing a rectangular graphite electrode, with nominal dimensions of 38 × 38 mm^2^. In Table 1, the workpiece chemical composition is presented, while in Table 2, the workpiece and electrode thermophysical properties are listed. The experiments were carried out on a Swiss-made Roboform Agie Charmilles 350Sp EDM; the experimental setup is graphically illustrated in Figure 1.

A full-scale experiment was conducted, with control parameters the pulse-on current and the pulse-on time, since, according to the literature [36,37,38,39], these machining parameters are mainly affecting the process. The Duty Factor was kept constant at 0.5, and square pulses of open and close circuit voltage 120 and 30 V respectively were utilized. Hydrocarbon oil was utilized as the dielectric fluid, which was properly channeled, in constant pressure, into the working tank for efficient debris flushing. The nominal cutting depth was set at 0.5 mm in order that the machined surface characteristics could be fully developed. Finally, in-between the experiments, the graphite electrode was being dried out, so that the actual electrode wear could be measured after the removal of the absorbed dielectric fluid. In Table 3, the experimental parameters in detail are listed. The MRR, TMRR, and TWR were calculated according to Equations (1)–(3) respectively:

(1) $$\mathrm{MRR}=\frac{W_{\mathrm{st}}-W_{\mathrm{fin}}}{t_{\mathrm{mach}}}\times \frac{1}{\rho_{w}}$$

(2) $$\mathrm{TMRR}=\frac{{\mathrm{El}}_{\mathrm{st}}-{\mathrm{El}}_{\mathrm{fin}}}{t_{\mathrm{mach}}}\times \frac{1}{\rho_{\mathrm{el}}}$$

(3) $$\mathrm{TWR}=\frac{{\mathrm{El}}_{\mathrm{st}}-{\mathrm{El}}_{\mathrm{fin}}}{W_{\mathrm{st}}-W_{\mathrm{fin}}}$$

with MRR in mm^3^/min, TMRR in mm^3^/min, TWR in gr/gr, ρ_w_ and ρ_el_ the workpiece and electrode material density respectively in gr/mm^3^, t_mach_ is the machining time in min, W_st_ and W_fin_ are the workpiece weights before and after the machining in gr, while El_st_ and El_fin_ are the electrode’s weights before and after the machining respectively in gr.

For the SR measurements, a 3D TOPO 01P contact profilometer was used, equipped with an induction measuring head with a diamond cone-shaped tip of 2 μm radius and 90° apex angle, while it has embodied a confocal sensor of 8 nm vertical resolution and 130 μm range. Based on the adopted norm of ISO 25178-2, the cut-off length was set at 8 mm, resulting in an evaluation length of 40 mm (5 times the cut-off length). For each measurement, 101 consecutive routes of 10 mm length were taken, with 0.5 mm/s measuring speed, resulting in a total scanned area of 1.25 × 10 mm^2^. The machined surfaces cross sections were polished and properly etched, in order the microstructural differences of the WL and the HAZ to be revealed and highlighted. The AWLT and the average HAZ thickness are calculated as the quotient of the respective area to the corresponding length.

**Figure 2:**
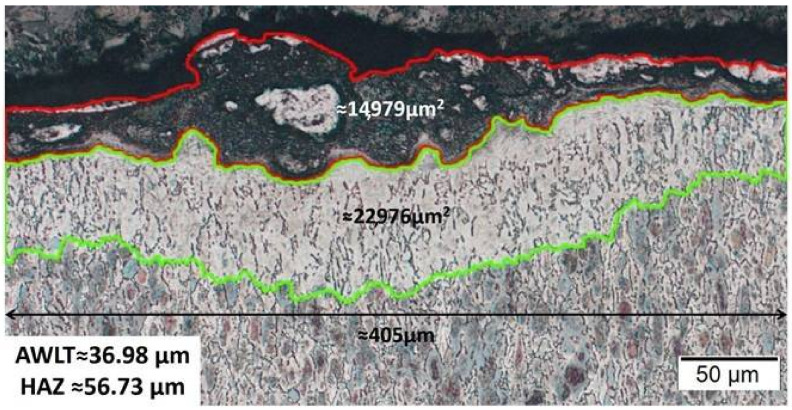
Machined surface cross section for I_p_ 65 A and T_on_ 100 μs, with highlighted the WL and HAZ areas.

In Figure 2, an example of the measuring method is presented, where the WL and the HAZ areas are 14,979 and 22,976 μm^2^ respectively, the corresponding length is 405 μm, and thus it is resulted an AWLT of 36.98 μm and a HAZ of 56.73 μm. Finally, the machined surfaces were studied through SEM microscopy, in order the surface integrity and the developed material formations to be observed on a microscale level. For all the aforementioned indexes (MRR, TMRR, TWR, Ra, Rt, AWLT, HAZ) ANOVA was performed, to define how they are affected by the machining parameters, namely the pulse-on current and time. Additionally, for the MRR and the TMRR, based on the RSM method, semi-empirical relations were developed and proposed, which correlate them with the machining parameters.

## 3. Results and Discussion

In Table 4 the experimental results are presented.

### 3.1. Material Removal Rate, Tool Material Removal Rate and Tool Wear Ratio

**Figure 3:**
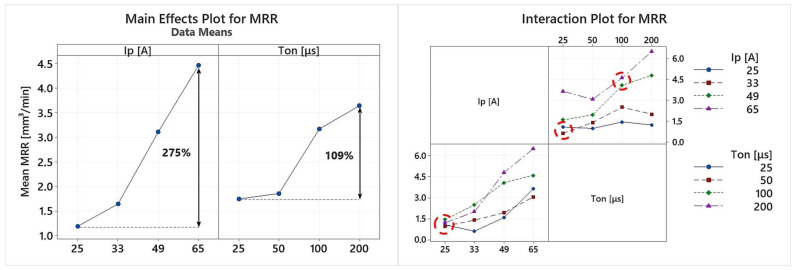
Main Effects Plot and Interaction Plot of MRR.

Intuitively it can be said that MRR and TWR strongly depend on the machining power and the per pulse energy, i.e., the pulse-on current and time. More intense machining parameters could lead in higher MRR, while the TWR could be also affected, since both the material removal rate and the electrode’s wear are related to these machining parameters. Nevertheless, and as it has been aforementioned, EDM is a complex multiparameter process, with no linear response, hence, such simplifications can only be used as a general principle, while an in-depth study is necessary. More specifically, the material removal is affected and limited, mainly, by three underlying physical mechanisms: the plasma channel growth, the debris concentration in-between the electrode and the workpiece, and the carbon decomposition. For higher pulse-on times, the plasma channel expands over time, by consuming significant amount of energy, while the energy density is degreased correspondingly [2,40,41]. At the same time, increased material removal rate results in a higher debris concentration in between the electrode and workpiece, which, from one point onwards, cannot be efficiently removed. Those debris may destabilize the process and/or cause arcing conditions, while, at the same time, machining power is spent on their re-melt [42]. Finally, decomposed carbon, coming from both the electrode (when a graphite electrode is utilized) and the dielectric fluid (when hydrocarbon oil is used), bonded on the surfaces, forming a “shield layer”, which, although it may act protectively for the tool electrode, may also be unbeneficial for the MRR [18]. The peculiar behavior of MRR and TWR during EDM machining has been reported in the literature [43,44], hence, it is important, scientifically interesting, and should be further investigated. In Figure 3, the Main Effect Plot and the Interaction Plot of MRR are presented.

From the Main Effects Plot of MRR, the general rule of thumb is that higher machining power and/or per-pulse energy lead in higher MRR is confirmed. Namely, as the pulse-on current increased from 25 to 65 A, the mean MRR increased too, approximately by 275%, from 1.19 to 4.46 mm^3^/min. Similarly, higher pulse-on times lead to higher mean MRR, with a 109% increase of mean MRR between 25 and 200 μs. At the same time, from a closer look on the Interaction Plot, some very interesting conclusions can be deduced. At first, we find that for 25 A the increase in T_on_ does not significantly affect the MRR, while for 25 and 33 A, as the pulse-on time increased from 100 to 200 μs, MRR slightly decreased. This “bizarre” behavior of MRR is in line with the literature results [38], and can be attributed to the complicated inherent underlying physical mechanisms, which were previously mentioned. One even more interesting observation is that for 25 μs pulse-on time, the MRR utilizing 25 A pulse-on current is greater than that of 33 A, while, at the same time, for 100 μs, 49 and 65 A result in almost the same MRR. This remark is of extreme interest since the pulse-on current is straight related with consuming power, and thus the cost of the process. It is concluded that there are combinations of machining conditions more suitable than others, which can obtain the same, even better, results with less power utilization and hence with less cost. Finally, and as it was expected, the higher MRR is achieved for 65 A pulse-on current and 200 μs pulse-on time.

Since MRR is one of the most important performance indexes, directly related with the machining efficiency and its economic feasibility, the possibility to be predicted based on the machining parameters of pulse-on current and time is extremely interesting and usable. By employing the RSM method, the semi-empirical relation of Equation (4), which correlates the machining parameters of pulse-on current and time with the MRR, is calculated.

(4) $$\mathrm{MRR}=-0.37+0.0171I_{p}+0.0087T_{\mathrm{on}}+261\cdot 10^{-6}I_{p}^{2}-73\cdot 10^{-6}T_{\mathrm{on}}^{2}+459\cdot 10^{-6}I_{p}T_{\mathrm{on}}$$

with MRR in mm^3^/min, I_p_ in A and T_on_ in μs.

**Figure 4:**
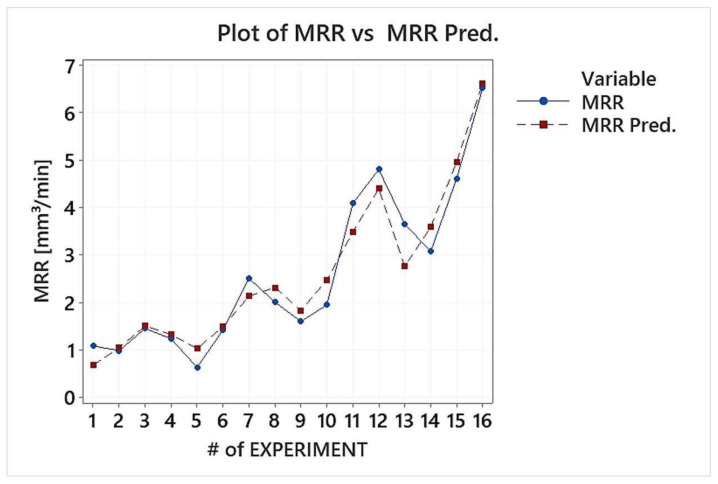
MRR and MRR predicted along with ANOVA results.

A model with linear, square, and interaction terms was adopted, resulting in an adequate predictability, and fitted with the experimental results. More specific, it has an R-sq and S values of 93.88% and 0.51 respectively, and an almost zero *p*-value. From the ANOVA (see Table 5) it is inferred that both the pulse-on current and time strongly affects the MRR, having a 61.65% and 21.94% contribution respectively, and close-to-zero *p*-values. Finally, the juxtaposition in Figure 4 of the experimental MRR values with predicted ones confirms that the current model properly describes the correlation between Ip, Ton, and MRR.

**Figure 5:**
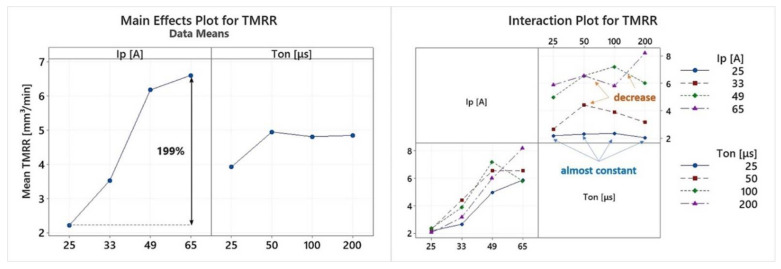
Main Effects Plot and Interaction Plot of TMRR.

In Figure 5, the Main Effects Plot and the Interaction Plot of the TMRR are presented, and some interesting conclusions can be deduced. As was expected, the pulse-on current strongly affects the TMRR, with its increase to result in higher TMRR. More specific, between 25 and 65 A, the mean TMRR increased by 199%. On the contrary, and a bit surprisingly, it seems that TMRR is not significantly affected by the pulse-on time, since the mean TMRR, after a slight increase between 25 and 50 μs, remained almost constant for pulse-on times up to 200 μs. Nevertheless, a more in-depth analysis is necessary based on the Interaction Plot. Indeed, increase in I_p_ leads to a higher TMRR for almost all the pulse-on times. On the other hand, higher T_on_ does not necessarily result in higher TMRR. In fact, for 25 A pulse-on currents, the increase of T_on_ does not significantly affect the TMRR, while in other cases, a higher T_on_ results lower TMRR. The pre-described peculiar behavior of TMRR lead us to conclude that the combination of the machining parameters is pivotal in EDM, and not each parameter by itself.

The predictability of TMRR could be most helpful, not only because it is directly linked with the machining cost, but also with the machining accuracy. The material removal from the electrode results in dimensional changes in electrodes, thus, the obtained accuracy is seriously affected. Hence, a relationship between the machining parameters and the TMRR could be useful for an efficient machining planning. Based on the RSM method, Equation (5) emerged, which correlates the I_p_, T_on_ and TMRR.

(5) $$T\mathrm{MRR}=-5.41+0.361I_{p}+0.0067T_{\mathrm{on}}-308\cdot 10^{-6}I_{p}^{2}-77\cdot 10^{-6}T_{\mathrm{on}}^{2}+332\cdot 10^{-6}I_{p}T_{\mathrm{on}}$$

with TMRR in mm^3^/min, I_p_ in A and T_on_ in μs.

**Figure 6:**
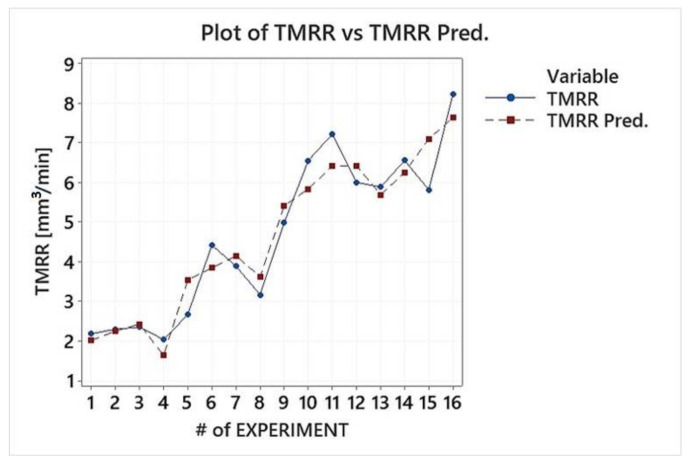
TMRR and TMRR Predicted along with ANOVA results.

Again, a model with linear, square, and interaction terms was employed, resulting an adequate accuracy of prediction. The R-sq and S values are 91.6% and 0.72 respectively, while the model’s *p*-Value is almost zero, which entails the model’s statistical significance (see Table 6). The ANOVA indicates that the most important and influencing parameter is the pulse-on current, having a 79% contribution, while the pulse-on time only has 1.26%. These results are in line and justified by the previous analysis. Finally, the plot of Figure 6 confirms the model’s fitness, since the predicted values are very close to the experimental ones.

**Figure 7:**
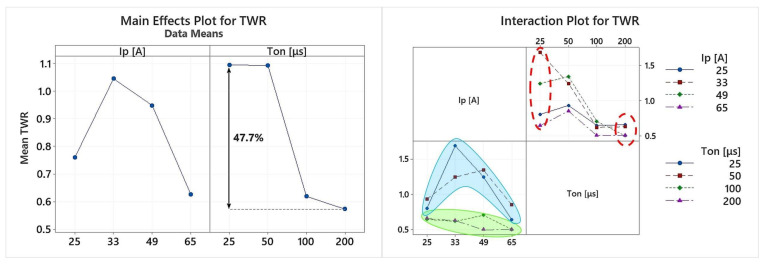
Main Effects Plot and Interaction Plot of TWR.

In Figure 7, the Main Effects Plot and the Interaction Plot of TWR are presented, allowing some interesting and helpful conclusions to be deduced. We find that TWR emerges from the superimposition of MRR and TMRR, and thus, it differs from both of them. For machining parameters that MRR and TMRR increase, TWR may decrease, since it is the percentage comparison of the electrode and workpiece wear. In more detail, although the TWR increased between 25 and 33 A, further increase in pulse-on current resulted in a decrease of TWR. On the other hand, higher pulse-on times resulted a reduced mean TWR, with a total decrease of 47.7% between 25 and 200 μs. As in MRR, the interaction plot gives us a clearer view of the process, and how the machining parameters affect each other. At first, for 100 and 200 μs pulse-on times, the TWR is not significantly affected by changes in pulse-on current (see Figure 7 green area), while for T_on_ 25 and 50 μs, the TWR strongly depends on the I_p_ (see Figure 7 blue area). Moreover, on the contrary, with the 25 μs where the TWR varies depending on the utilized pulse-on current, for 200 μs the TWR does not seem notably affected by the pulse-on current. Hence, it is deduced that there are some preferable combinations of machining parameters, which result in a lower TWR, and thus, lower machining costs, making the machining economically feasible. However, in machining planning, there are other criteria that have also to be considered, like the SR and the surface quality, parameters that will be discussed below.

### 3.2. Surface Roughness and Surface Quality

Surface Roughness and Surface Quality are essential parameters in EDM, and have always to be considered during machining planning, since they are directly related with a components’ functionality. In cases where EDM is the final process, obviously, the manufactured parts have to meet some predefined quality standards. At the same time, if a post-process is necessary, especially when high power EDM is utilized as roughing machining, it is crucial for the surface characteristics to be known, in order that the subsequent process can be planned properly. Hence, the surface study after EDM, besides being of academic interest, is also important in practice.

**Figure 8:**
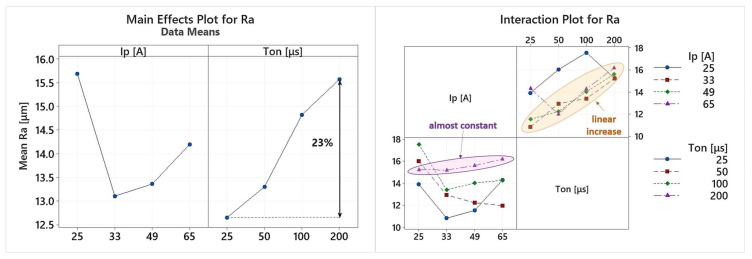
Main Effects Plot and Interaction Plot of Ra.

As it was previously described, with each spark an amount of material is removed by the workpiece, leaving behind a tiny crater. The total material removal is the accumulative result of thousands or millions of successive sparks. The obtained SR is apparently linked with the formed craters morphological characteristics, which, according to the literature, depends on the machining parameters. As a general rule, the crater volume is impacted by the pulse energy, with the pulse-on current mostly affecting the crater depth, and the pulse-on time its width [2]. However, the final SR is far more complicated, because of the stochastic nature of EDM, the superimposition of successive craters, and the formation of the White Layer. Conceptually, EDM is a chaotic process in micro-scale (spatial and time), thus, any attempt for a strictly deterministic interpretation would be deficient [45]. This observation entails that the sparks, to some extent, are being formed randomly, hence, the SR does not come from ordered craters, but mostly of randomly overlapped ones. Finally, by the material that melts on each spark, only a proportion is removed, with the rest being re-solidified on a workpiece surface. At the same time, ablated material that remains proximal to the surface may be re-condensed and adhered on it, forming “debris adheres” over the surface. The re-solidified and re-condensed material form an amorphous layer, best known as a White Layer, with more distinctive properties than the mother material. The WL properties (thickness, and morphological characteristics) mainly depend on the machining parameters (i.e., pulse-on current and time), the electrode and workpiece material and the utilized dielectric fluid. On the WL, different formations can be observed such as crater marks, uneven depositions of melted and re-solidified material that form islets, scattered debris, inclusions, pockmarks, and cracks. The cracks are incurred due to the combined effect of existing residual stresses, and the induced thermal ones, while the developing high gradients in temperature and pressure favor their formation [2,38]. In Figure 8, the Main Effects Plot and the Interaction Plot of Ra are presented.

By the ANOVA plots of Ra, it is confirmed that there is an effect of machining parameters on the Ra. Although a decrease in mean Ra is observed between 25 and 33 A, further increase of I_p_ resulted in higher mean Ra. Furthermore, the increase of mean Ra in respect of the increase in pulse-on time is almost linear, having an approximately 23% rise between 25 and 200 μs. Regarding the Interaction Plot, two comments have to be made; at first, the positive linear correlation between Ra and T_on_ is significantly strong for 33 and 49 A (see Figure 8 orange area), while 25 and 65 A (the outliers) have a more vague behavior. Moreover, for 200 μs pulse-on current the Ra remains nearly steady for all the pulse-on currents.

**Figure 9:**
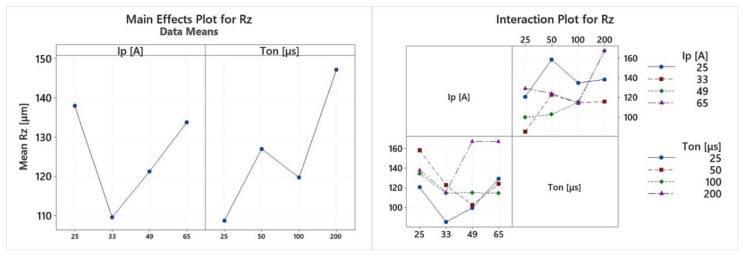
Main Effects Plot and Interaction Plot of Rz.

Contrary to the Ra, Rz does not seem to have a specific correlation with the pulse-on current and time. From the Main Effects Plot and the Interaction Plot of Figure 9 it is deduced that higher pulse-on current and/or time do not compulsorily lead to higher Rz. For example, the Rz for 33 A is always lower than those of 25 A, while for 65 A the lowest Rz was measured for T_on_ 100 μs. This vague and ambiguous behavior of Rz can be interpreted and rendered to the underlying mechanisms of EDM and the principles in measuring Rz. Conceptually the Rz refers to the maximum peak to valley height of a given profile. In EDM, there are such intense conditions of temperature and pressure that being topically developed, random re-depositions of molten and ablated material may easily increase the Rz, and although a measuring norm is followed (ISO 25178-2), a degree of randomness is inevitable. Hence, a strict conclusion regarding the correlation between Rz and pulse-on current and time, especially for such intense machining parameters, is considered precarious. On the other hand, and since Ra, by its definition, consists of an arithmetical mean deviation of the assessed profile, it is reasonable to follow specific trends, as was previously analyzed.

**Figure 10:**
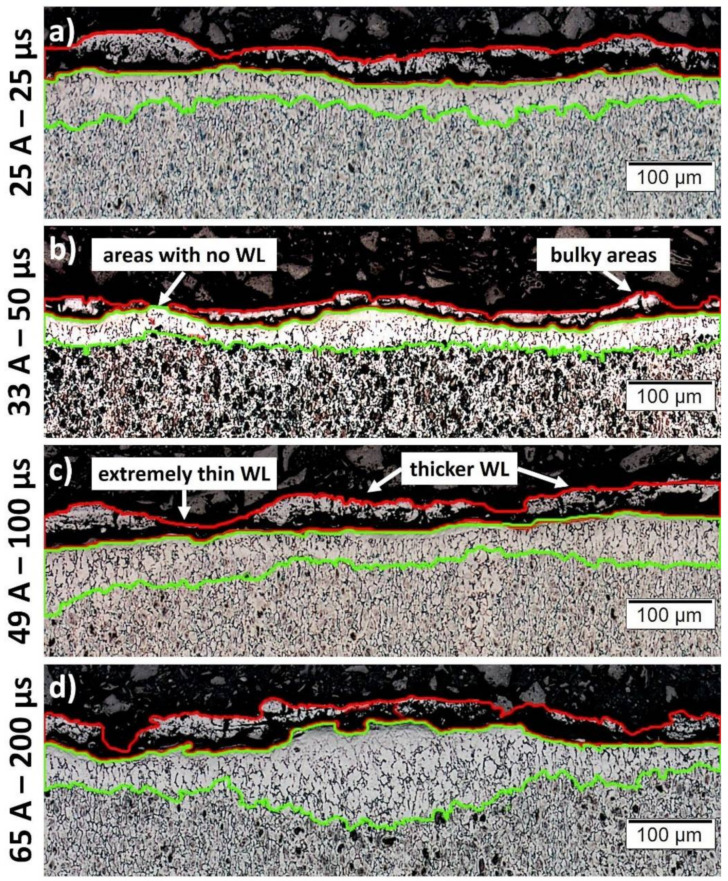
Cross sections where the WL and the HAZ can be observed and measured for (**a**) 25 A—25 μs, (**b**) 33 A—50 μs, (**c**) 49 A—100 μs and (**d**) 65 A—200 μs.

In Figure 10, the cross sections for different machining parameters are depicted, where the WLs and the HAZs are clearly distinguished.

The gradual change in WL characteristics as higher machining power and per-pulse energy are utilized can be observed and qualitatively assessed. For 25 A and 25 μs pulse-on current and time respectively, the WL is relatively thick, with a high degree of uniformity. For I_p_ 33 A and T_on_ 50 μs the AWLT significantly decreased, acquiring inhomogeneity, resulting in some more bulky areas and areas with no WL. For more intense machining parameters (i.e., 49 A and 100 μs), the WL regained its thickness, but retained its inhomogeneity, thus, there are areas with a major difference in their WL thickness (areas with extremely thin WL and areas with thicker WL). Finally, for 65 A and 200 μs the WL follows the aforementioned trend to become thicker and more irregular. On the other hand, the HAZ seems to be less sensitive to changes in machining parameters. It is mainly evenly spread underneath the WL, and only for 65 A and 200 μs is a degree of unevenness acquired.

**Figure 11:**
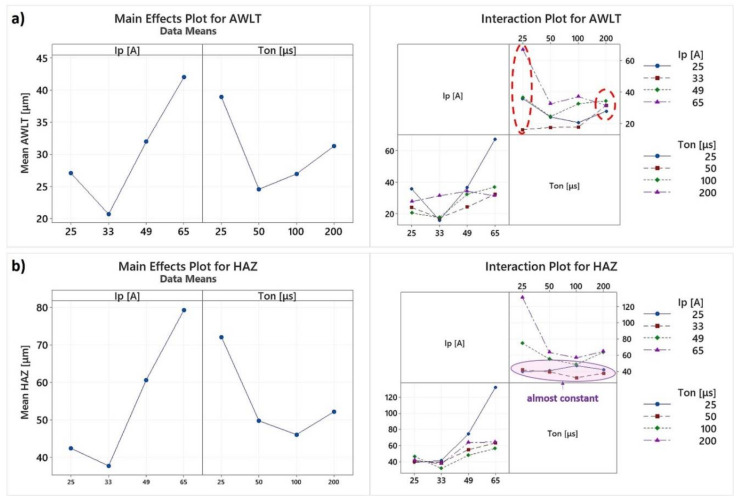
Main Effects Plot and the Interaction Plot of (**a**) AWLT and (**b**) HAZ.

In Figure 11, the Main Effects Plot and the Interaction Plot of AWLT and HAZ are presented, and hence, a more quantitative evaluation can be done. At first, we see that the mean values of AWLT and HAZ follow the same tendency, namely, they decrease between 25 and 33A and after that they increase. This behavior becomes even more interesting since the mean Ra also follows this pattern, implying and confirming that AWLT is correlated with Ra. On the other hand, the pulse-on time has a vaguer effect on the AWLT and the HAZ, something that is further corroborated by the Interaction Plots. For 25 μs T_on_, the AWLT and the HAZ rapidly change in respect to the utilized I_p_, while, for the rest pulse-on times the variation is significantly smoother as the pulse-on current changes. Moreover, for 33 and 49 A the HAZ remains almost constant for all the pulse-on time. Finally, a quite interesting remark is that the AWLT for 200 μs is not notably impacted by changes in pulse-on current, on the contrary with the 25 μs T_on_, where the AWLT is highly affected by the pulse-on current.

**Figure 12:**
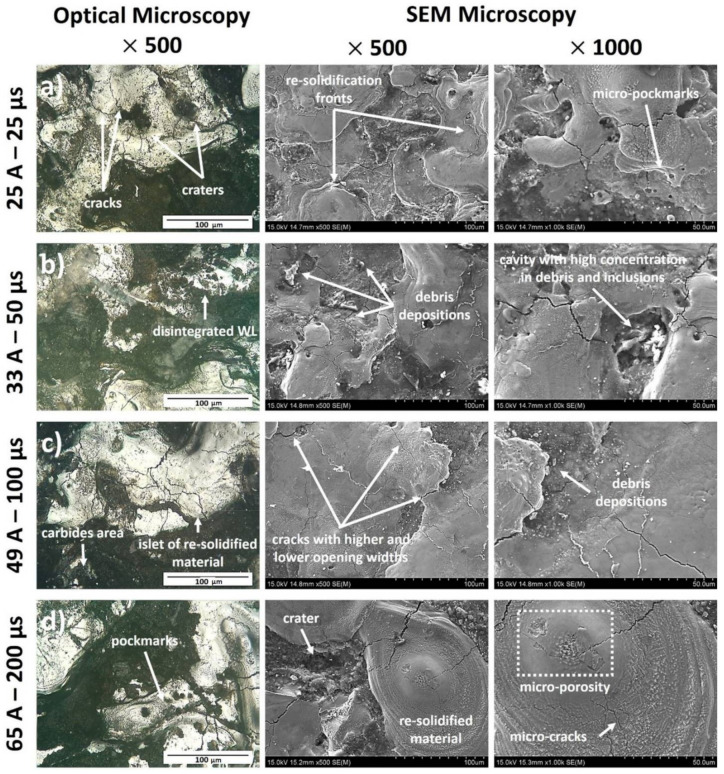
Images from optical and SEM microscopy for (**a**) 25 A—25 μs, (**b**) 33 A—50 μs, (**c**) 49 A—100 μs, and (**d**) 65 A—200 μs.

Closing the surface characterization section, the machined surfaces were observed through optical and SEM microscopy. In Figure 12 images from an optical and SEM microscope are presented, for different machining powers and per-pulse energies, where the typical formations of the EDMed surfaces can be distinguished. Namely, for 25 A and 25 μs, the surface is largely covered by a thick WL, with cracks running it through. Moreover, in SEM images, the re-solidification fronts can be observed, while, in further magnification, some micro-pockmarks can be remarked. The dark areas are reasonably believed to be carbides [44,46], which are formed under the high temperature and pressure conditions that are developed during machining. In the 33 A and 50 μs, the WL has been reduced, covering a smaller percentage of the total surface. Along with some bulky WL formations, there are also thinner ones (almost disintegrated), a fact that has been already pointed out during the analysis of the cross sections (see Figure 10). In SEM images, debris depositions, and cavities filled with debris and other inclusions are shown as well. At 49 A and 100 μs the surface is again largely covered by a WL, which forms islets of re-solidified material.

Cracks with higher and lower opening widths are present (micro- and macro- cracks), along with debris depositions. Finally, for 65 A and 200 μs, the surface is heterogeneous, covered with bulky areas of WL and others of carbides. Moreover, wide cracks and big pockmarks are spread across the surface, while on the re-solidified material areas micro-porosity and micro-cracks were developed. The crack’s density increases in respect of the machining power and the per-pulse energy, with the cracks acquiring wider opening widths and greater depths, penetrating sometimes the whole WL thickness. The characteristics of the machined surface is of extreme interest and importance, since they are straight related with the machined parts of mechanical strength properties, hence during machining planning they have always to be considered.

## 4. Conclusions

In the current study, a comprehensive experimental investigation regarding the processing of Ti–6Al–4V ELI with a high power EDM by using a graphite electrode was presented. A full-scale experiment was carried out, with control parameters the pulse-on current and time, which varied from 25 up to 65 A and from 25 up to 200 μs respectively. The machining efficiency and feasibility were estimated according to the Material Removal Rate (MRR), the Tool Material Removal Rate (TMRR), and the Tool Wear Ratio (TWR), while the Surface Roughness was evaluated in terms of Ra and Rz. Moreover, the machined surfaces were observed through optical and SEM microscopy, in order that the surface characteristics could be studied, as well their cross sections, so that the AWLT and the HAZ thickness could be measured. For the aforementioned performance indexes, an ANOVA was performed, in order to be adequately understood how process parameters impact the machining result. Finally, for the MRR and TMRR, based on the RSM method, semi-empirical relations were proposed, correlating them with the pulse-on current and time. The current study deduced the following main conclusions:

- The MRR is affected by both, the pulse-on current and time, although the pulse-on current has a greater impact on it. This conclusion is not entailed only by the ANOVA analysis, but from the RSM model as well, in which the contribution of I_p_ is significantly higher than that of T_on_.
- The TMRR mainly depends on the pulse-on current, while the pulse-on time has a minor and a vague effect on it. Moreover, in the RSM model, the I_p_ term is an order of magnitude of higher significance that T_on_.
- The TWR strongly depends on the machining parameters combination, with some to be, in terms of TWR, far more preferable than others.
- The mean Ra is increased in respect of the pulse-on time, while the pulse-on current affects it in a more fuzzy and ambiguous way. On the other hand, for the Rz, any strict correlation with I_p_ and T_on_ would be precarious, at least for those machining conditions, since a clear trend or pattern cannot be deduced.
- The WL characteristics significantly change depending on the machining conditions. The WL thickness and its homogeneity are altered according to the machining power and the per-pulse energy that is utilized. On the contrary, the HAZ seems to be less sensitive in changes in the machining parameters.
- Through the microscopy (optical and SEM), typical formations of the EDMed surfaces were depicted. Namely, cracks with different opening widths and depths, craters, re-solidified material that forms islets, debris and carbides depositions, pockmarks, as well areas with developed micro-porosity, were observed. These surface characteristics are varied according to the machining conditions.

## Figures and Tables

**Table 1:** Titanium Grade 23 ELI chemical composition.

Ti	C max (%)	Fe max (%)	H max (%)	N max (%)	O max (%)	V (%)	Al (%)
Bal.	0.08	0.25	0.0125	0.03	0.13	3.5–4.5	5.5–6.5

**Table 2:** Mechanical properties of Titanium Grade23 ELI and Graphite electrode.

Material	Graphite	Ti Grade 23 ELI
Density (g/cm^3^)	1.77	4.43
Melting Point (°C)	3300	1600
Electrical Resistively (μΩ cm^−1^)	1400	53.3
Hardness (HB)	7	326
Thermal Conductivity (W/mK)	168	16.70

**Table 3:** Experimental Parameters.

Machining Conditions	Level 1	Level 2	Level 3	Level 4
Pulse-on Current (A)	25	33	49	65
Pulse-on Time (μs)	25	50	100	200
Duty Factor	0.5			
Polarity	Straight			
Waveform	Square pulses			
Open Circuit Voltage (V)	120			
Close Circuit Voltage (V)	30			
Dielectric	Synthetic Hydrocarbon Fluid			
Dielectric Flushing	Side Flushing with pressure			
Dielectric Flushing Pressure (MPa)	0.7 (Constant)			
Recoil	0.2 μs per 0.6 μs with speed of 1000 mm/min			

**Table 4:** Experimental results.

#	Ip (A)	Ton (μs)	MRR (mm^3^/min)	TMRR (mm^3^/min)	TWR	Ra (μm)	Rz (μm)	AWLT (μm)	HAZ (μm)
1	25	25	1.08	2.18	0.80	13.9	120.6	35.79	39.60
2	25	50	0.98	2.29	0.93	16.0	158.4	24.05	40.86
3	25	100	1.45	2.35	0.65	17.6	134.6	20.57	47.00
4	25	200	1.23	2.03	0.66	15.2	138.2	27.80	42.16
5	33	25	0.63	2.66	1.69	10.8	85.3	16.00	41.81
6	33	50	1.42	4.41	1.24	12.9	122.8	17.44	39.34
7	33	100	2.51	3.88	0.62	13.4	114.6	17.75	32.07
8	33	200	2.00	3.16	0.63	15.2	115.7	31.49	37.70
9	49	25	1.60	4.98	1.25	11.5	99.7	36.63	74.93
10	49	50	1.95	6.54	1.34	12.2	102.8	24.37	55.27
11	49	100	4.09	7.21	0.70	14.0	115.1	32.46	48.47
12	49	200	4.80	6.00	0.50	15.6	167.4	34.35	63.93
13	65	25	3.65	5.87	0.64	14.3	129.2	67.14	132.00
14	65	50	3.07	6.55	0.85	12.0	124.1	32.36	63.49
15	65	100	4.61	5.81	0.50	14.3	114.4	36.99	56.73
16	65	200	6.51	8.21	0.50	16.2	167.4	31.41	64.85

Note: # represents the number of the experiment.

**Table 5:** Analysis Of Variance for MRR.

Analysis of Variance							
Source	DF	Seq SS	Contribution	Adj SS	Adj MS	F-Value	*p*-Value
Model	5	40.2865	93.88%	40.2865	8.0573	30.69	0
Linear	2	35.8677	83.58%	39.9774	19.9887	76.13	0
I_p_(A)	1	26.4539	61.65%	29.7724	29.7724	113.4	0
T_on_(A)	1	9.4138	21.94%	11.4278	11.4278	43.53	0
Square	2	0.8489	1.98%	0.8489	0.4244	1.62	0.246
I_p_ (A)·I_p_ (A)	1	0.0302	0.07%	0.0302	0.0302	0.11	0.742
T_on_ (μs) T_on_ (μs)	1	0.8187	1.91%	0.8187	0.8187	3.12	0.108
2-Way Interaction	1	3.5699	8.32%	3.5699	3.5699	13.6	0.004
I_p_ (A) ·T_on_ (μs)	1	3.5699	8.32%	3.5699	3.5699	13.6	0.004
Error	10	2.6255	6.12%	2.6255	0.2626	-	-
Total	15	42.912	100.00%	-	-	-	-

**Table 6:** Analysis Of Variance for TMRR.

Analysis of Variance							
Source	DF	Seq SS	Contribution	Adj SS	Adj MS	F-Value	*p*-Value
Model	5	56.6081	91.60%	56.6081	11.3216	21.81	0
Linear	2	49.6418	80.33%	51.9521	25.9761	50.04	0
I_p_(A)	1	48.8631	79.07%	51.0921	51.0921	98.42	0
T_on_(A)	1	0.7786	1.26%	1.3757	1.3757	2.65	0.135
Square	2	5.0933	8.24%	5.0933	2.5467	4.91	0.033
I_p_ (A)·I_p_ (A)	1	4.2039	6.80%	4.2039	4.2039	8.1	0.017
T_on_ (μs) ·T_on_ (μs)	1	0.8894	1.44%	0.8894	0.8894	1.71	0.22
2-Way Interaction	1	1.873	3.03%	1.873	1.873	3.61	0.087
I_p_ (A) ·T_on_ (μs)	1	1.873	3.03%	1.873	1.873	3.61	0.087
Error	10	5.191	8.40%	5.191	0.5191	-	-
Total	15	61.7991	100.00%	-	-	-	-

## Data Availability

The data presented in this study are available in Karmiris-Obratański, P.; Papazo-glou, E.L.; Leszczyńska-Madej, B.; Zagórski, K.; Markopoulos, A.P. A comprehensive study on processing Ti–6Al–4V ELI with high power EDM.

## Nomenclature

EDM	Electrical Discharge Machining	
AWLT	Average White Layer Thickness	μm
E_fin_	Electrode weight after machining	gr
E_st_	Electrode weight before machining	gr
HAZ	Heat Affected Zone	μm
I_p_	Pulse-on current	A
MRR	Material Removal Rate	mm^3^/min
Ra	Mean Roughness	μm
Rz	Maximum peak to valley height	μm
SCD	Surface Crack Density	m/mm^2^
SQ	Surface Quality	
ST	Surface Topography	
TMRR	Tool Material Removal Rate	mm^3^/min
T_on_	Pulse-on time	μs
TWR	Tool Wear Ratio	%
t_mach_	Mahining time	min
W_fin_	Workpiece weight after machining	gr
W_st_	Workpiece weight before machining	gr
WL	White Layer	
ρ_el_	Electrode density	gr/mm^3^
ρ_w_	Workpiece density	gr/mm^3^
DF	Degrees of freedom	
Seq SS	Sequential sums of squares	
Adj SS	Adjusted sums of squares	
Adj MS	Adjusted mean squares	

## References

[1] Raju L., Hiremath S.S. (2016). A State-of-the-art Review on Micro Electro-discharge Machining. Procedia Technol..

[2] Jahan M.P. (2015). Electrical Discharge Machining (EDM): Types, Technologies and Applications.

[3] Fonda P., Wang Z., Yamazaki K., Akutsu Y. (2008). A fundamental study on Ti–6Al–4V’s thermal and electrical properties and their relation to EDM productivity. J. Mater. Process. Technol..

[4] Gostimirović M., Kovac P., Sekulic M., Skoric B. (2012). Influence of discharge energy on machining characteristics in EDM. J. Mech. Sci. Technol..

[5] Gao C., Liu Z. (2003). A study of ultrasonically aided micro-electrical-discharge machining by the application of workpiece vibration. J. Mater. Process. Technol..

[6] Marafona J.D., Chousal J. (2006). A finite element model of EDM based on the Joule effect. Int. J. Mach. Tools Manuf..

[7] Faisal N., Kumar K. (2018). Optimization of Machine Process Parameters in EDM for EN 31 Using Evolutionary Optimization Techniques. Technologies.

[8] Jameson E.C. (2001). Electrical Discharge Machining.

[9] Lin Y.-C., Chen Y.-F., Wang D.-A., Lee H.-S. (2009). Optimization of machining parameters in magnetic force assisted EDM based on Taguchi method. J. Mater. Process. Technol..

[10] Ho S., Aspinwall D., Voice W. (2007). Use of powder metallurgy (PM) compacted electrodes for electrical discharge surface alloying/modification of Ti–6Al–4V alloy. J. Mater. Process. Technol..

[11] Nikalje A.M., Kumar A., Srinadh K.V.S. (2013). Influence of parameters and optimization of EDM performance measures on MDN 300 steel using Taguchi method. Int. J. Adv. Manuf. Technol..

[12] Ho K.H., Newman S.T. (2003). State of the art electrical discharge machining (EDM). Int. J. Mach. Tools Manuf..

[13] Soni J., Chakraverti G. (1996). Experimental investigation on migration of material during EDM of die steel (T215 Cr12). J. Mater. Process. Technol..

[14] Erden A. (1983). Effect of Materials on the Mechanism of Electric Discharge Machining (E.D.M.). J. Eng. Mater. Technol..

[15] Kruth J.-P., Stevens L., Froyen L., Lauwers B. (1995). Study of the White Layer of a Surface Machined by Die-Sinking Electro-Discharge Machining. CIRP Ann..

[16] Kaneko T., Tsuchiya M. (1988). Three-dimensional numerically controlled contouring by electric discharge machining with compensation for the deformation of cylindrical tool electrodes. Precis. Eng..

[17] Abu Qudeiri J., Mourad A.-H.I., Ziout A., Abidi M.H., Elkaseer A. (2018). Electric discharge machining of titanium and its alloys: Review. Int. J. Adv. Manuf. Technol..

[18] Manjaiah M., Narendranath S., Basavarajappa S. (2014). A review on machining of titanium based alloys using EDM and WEDM. Rev. Adv. Mater. Sci..

[19] Davim J.P. (2014). Machining of Titanium Alloys.

[20] Klocke F., Zeis M., Klink A., Veselovac D. (2012). Technological and Economical Comparison of Roughing Strategies via Milling, EDM and ECM for Titanium- and Nickel-based Blisks. Procedia CIRP.

[21] Lin Y.C., Yan B.H., Chang Y.S. (2000). Machining characteristics of titanium alloy (Ti–6Al–4V) using a combination process of EDM with USM. J. Mater. Process. Technol..

[22] Hasçalık A., Çaydaş U. (2007). Electrical discharge machining of titanium alloy (Ti–6Al–4V). Appl. Surf. Sci..

[23] Hasçalık A., Çaydaş U. (2007). A comparative study of surface integrity of Ti–6Al–4V alloy machined by EDM and AECG. J. Mater. Process. Technol..

[24] Kumar S., Batish A., Singh R., Singh T.P. (2014). A hybrid Taguchi-artificial neural network approach to predict surface roughness during electric discharge machining of titanium alloys. J. Mech. Sci. Technol..

[25] Wang X., Liu Z., Xue R., Tian Z., Huang Y. (2014). Research on the influence of dielectric characteristics on the EDM of titanium alloy. Int. J. Adv. Manuf. Technol..

[26] Mower T.M. (2014). Degradation of titanium 6Al–4V fatigue strength due to electrical discharge machining. Int. J. Fatigue.

[27] Khan A.R., Rahman M., Salehina Z.U., Rahman S. (2016). Optimal set-up and surface finish characteristics in electrical discharge machining on Ti-5Al-2.5Sn using graphite. Perspect. Sci..

[28] Khan A.R., Rahman M. (2017). Surface characteristics of Ti-5Al-2.5Sn in electrical discharge machining using negative polarity of electrode. Int. J. Adv. Manuf. Technol..

[29] Nair S., Dutta A., Giridharan A. (2019). Investigation on EDM machining of Ti6Al4V with negative polarity brass electrode. Mater. Manuf. Process..

[30] Prakash C., Kansal H.K., Pabla B.S., Puri S. (2017). Experimental investigations in powder mixed electric discharge machining of Ti–35Nb–7Ta–5Zrβ-titanium alloy. Mater. Manuf. Process..

[31] Ahmed N., Ishfaq K., Moiduddin K., Ali R., Al-Shammary N. (2018). Machinability of titanium alloy through electric discharge machining. Mater. Manuf. Process..

[32] Ahmed N., Anwar S., Ishfaq K., Rafaqat M., Saleh M., Ahmad S. (2019). The potentiality of sinking EDM for micro-impressions on Ti-6Al-4V: Keeping the geometrical errors (axial and radial) and other machining measures (tool erosion and work roughness) at minimum. Sci. Rep..

[33] Farooq M.U., Mughal M.P., Ahmed N., Mufti N.A., Al-Ahmari A., He Y. (2020). On the Investigation of Surface Integrity of Ti6Al4V ELI Using Si-Mixed Electric Discharge Machining. Materials.

[34] Bui V.D., Mwangi J.W., Schubert A. (2019). Powder mixed electrical discharge machining for antibacterial coating on titanium implant surfaces. J. Manuf. Process..

[35] Sen I., Karthikeyan G., Ramkumar J., Balasubramaniam R. (2012). A Study on Machinability of B-Modified Ti-6Al-4V Alloys by EDM. Mater. Manuf. Process..

[36] Lee H.T., Yur J.P. (2000). Characteristic Analysis of EDMed Surfaces Using the Taguchi Approach. Mater. Manuf. Process..

[37] Jabbaripour B., Sadeghi M.H., Faridvand S., Shabgard M.R. (2012). Investigating the Effects of Edm Parameters on Surface Integrity, Mrr and Twr in Machining of Ti–6Al–4V. Mach. Sci. Technol..

[38] Kushwaha A., Jadam T., Datta S., Masanta M. (2019). Assessment of Surface Integrity During Electrical Discharge Machining of Titanium Grade 5 Alloys (Ti-6Al-4V). Mater. Today Proc..

[39] Karmiris-Obratański P., Zagórski K., Cieślik J., Papazoglou E.L., Markopoulos A. (2020). Surface Topography of Ti 6Al 4V ELI after High Power EDM. Procedia Manuf..

[40] Shabgard M., Ahmadi R., Seyedzavvar M., Oliaei S.N.B. (2013). Mathematical and numerical modeling of the effect of input-parameters on the flushing efficiency of plasma channel in EDM process. Int. J. Mach. Tools Manuf..

[41] Papazoglou E.L., Markopoulos A., Papaefthymiou S., Manolakos D.E. (2019). Electrical discharge machining modeling by coupling thermal analysis with deformed geometry feature. Int. J. Adv. Manuf. Technol..

[42] Verma V., Sahu R. (2017). Process parameter optimization of die-sinking EDM on Titanium grade–V alloy (Ti6Al4V) using full factorial design approach. Mater. Today Proc..

[43] Verma V., Sajeevan R. (2015). Multi Process Parameter Optimization of Diesinking EDM on Titanium Alloy (Ti6Al4V) Using Taguchi Approach. Mater. Today Proc..

[44] Holsten M., Koshy P., Klink A., Schwedt A. (2018). Anomalous influence of polarity in sink EDM of titanium alloys. CIRP Ann..

[45] Aich U. (2018). Investigation for the presence of chaos in surface topography generated by EDM. Tribol. Int..

[46] Kolli M., Kumar A. (2015). Effect of dielectric fluid with surfactant and graphite powder on Electrical Discharge Machining of titanium alloy using Taguchi method. Eng. Sci. Technol. Int. J..

